# The Effects of Compound Chinese Herbal Medicine on the Growth and Digestive and Immune Systems of *Megalobrama amblycephala*

**DOI:** 10.3390/ani16060925

**Published:** 2026-03-15

**Authors:** Xijing Ye, Yunsheng Zhang, Hu Xia, Huangjie Fan, Jiahui Hu, Yanan Gong, Rurou Fu, Fuyan Chen, Liangguo Liu

**Affiliations:** 1 State Key Laboratory of Development Biology of Freshwater Fish Sub-Center for Health Aquaculture, College of Life and Environmental Sciences, Hunan University of Arts and Science, Changde 415000, China; yexijing1014@126.com (X.Y.); fhuangjie@126.com (H.F.); hujiahui060809@126.com (J.H.); llg1818@126.com (L.L.); 2 Key Laboratory of Aquaculture Genetic and Breeding and Healthy Aquaculture of Guangxi, Guangxi Academy of Fishery Sciences, Nanning 530021, China; fuyan7386@163.com; 3 Key Laboratory of Featured Aquaculture, Hunan Applied and Technology University, Changde 415000, China; ynangong@126.com (Y.G.); Fu19173609473@126.com (R.F.)

**Keywords:** *Megalobrama amblycephala*, compound Chinese herbal medicine, non-specific immunity, growth indexes

## Abstract

Chinese herbal medicine is rich in active ingredients that can promote growth and enhance immune function. In this study, *Lycium barbarum*, *Panax ginseng*, *Astragalus membranaceus* and *Phragmitis rhizoma* were crushed and mixed to prepare a compound Chinese herbal medicine. The basic feed of *Megalobrama amblycephala* was supplemented with 0 (control group), 1% (T1), 2% (T2) and 4% (T3) of this compound medicine. After raising for 90 days, in the T1 and T2 experimental groups, the length and width of intestinal villi and the activities of amylase, trypsin and lipase in the intestine were significantly higher than those in the control group. The weight gain rate and specific growth rates were highest and the feed coefficient was lowest in the T2 experimental group. In the control group, a large number of dilated hepatic sinusoids were detected, while this number significantly decreased in the T1 experimental group and they were not detected at all in the T2 and T3 experimental groups. The spleen and liver body indices were highest in the T2 experimental group. In all experimental groups, the Lys content and the activities of T-SOD, CAT, ACP, AKP and GSH-PX in serum were significantly higher than those of the control group. The expression of IgM, C3, TNF-ɑ and IL-1β in the head kidney; C3, TNF-ɑ and IL-1β in the spleen; C3 and IL-1β in the gills; IgM, C3 and IL-1β in liver; and IL-1β in the intestine was highest in the T2 experimental group. After challenge with *Aeromonas hydrophila*, the cumulative mortality rate of *M. amblycephala* was lowest in the T2 experimental group. The results of this study indicated that this compound Chinese herbal medicine could significantly enhance immunity, increase the activity of intestinal digestion-related enzymes and promote the growth of *M. amblycephala*. The appropriate addition amount of this compound Chinese herbal medicine in the basic feed of *M. amblycephala* was 2%.

## Simple Summary

As a natural and environmentally friendly feed additive, compound Chinese herbal medicine has a significant promoting effect on the growth of fish and enhances immunity. In this study, four Chinese herbal medicines, *Lycium barbarum*, *Astragalus membranaceus*, *Panax ginseng* and *Rhizoma phragmitis*, were prepared into a compound Chinese herbal medicine, which was added to the basic feed of *Megalobrama amblycephala* for breeding experiments to explore its effects on the growth, immunity and digestive function of this species. The aim of this was to provide a valuable basis for the sustainable and healthy breeding of *Megalobrama amblycephala*. The results of this study indicated that this compound Chinese herbal medicine could significantly enhance immunity, increase the activity of intestinal digestion-related enzymes and promote the growth of *Megalobrama amblycephala*. The appropriate addition amount of this compound Chinese herbal medicine in the basic feed of *Megalobrama amblycephala* was 2%.

## 1. Introduction

Blunt snout bream *(Megalobrama amblycephala*), commonly known as Wuchang fish, is a unique freshwater-cultured fish in China. It has the characteristics of rapid growth, strong resistance and delicate meat, and it is deeply appreciated by consumers. Since the promotion of its culture in 1960, *M. amblycephala* has become the sixth largest freshwater-cultured fish in China, and it is cultivated in all provinces except Xizang Autonomous Region and Qinghai. Its output was about 800,000 tons in 2023. With increases in both production and the degree of intensive farming, the growth rate of *M. amblycephala* has slowed, with the frequent occurrence of fish diseases becoming the main factor restricting the farming of healthy *M. amblycephala*. At present, chemicals and antibiotics are used in aquaculture to prevent the outbreak of fish diseases. This practice not only affects the quality and safety of aquatic products but also causes environmental problems, such as ecological damage. This has seriously hindered the sustainable development of aquaculture. In response to these problems, the Ministry of Agriculture and Rural Affairs issued Announcement No. 194: “From 1 January 2020, all kinds of growth-promoting drug feed additives except traditional Chinese medicine will be phased out” [1]. A complete ban on antibiotics has been implemented nationwide and ecological breeding has become mainstream; thus, new types of green and environmentally friendly feed additives are in urgent need of development.

Chinese herbal medicine has a long history and there are abundant resources in China. It also has the advantages of naturalness, safeness and lack of residues [2]. *Lycium barbarum* is a traditional Chinese medicine that is both edible and medicinal [3]. Its main active component, polysaccharides, has an enticing effect on carnivorous freshwater fish, crabs, etc. Long-term feeding in aquaculture can effectively reduce the feed coefficient [3]. *Astragalus membranaceus* can eliminate hydroxyl radicals and superoxide anion radicals, and adding an appropriate amount of fermented *Astragalus membranaceus* to feed was found to enhance the antioxidant index of *Epinephelus fuscoguttatus* [4]. *Panax ginseng* also contains many beneficial active ingredients, and its polysaccharides and ginsenosides are widely used in aquaculture, with the former able to significantly improve the weight gain rate and liver body index in *Sparus inacrocephalus* [5,6]. Ginsenoside has been proven to have anti-inflammatory effects, and the traditional Chinese medicine Shengmai drink, prepared from ginsenoside, might weaken the expression of pro-inflammatory cytokines [7]. Reed root is the fresh or dried rhizome of poaceae plant reeds. Water extracts of this root can alleviate lung tissue fibrosis and damage in mice with radiation-induced lung injury, as well as inhibit lung cell apoptosis and inflammatory responses [8,9]. As a green and environmentally friendly feed additive, the application of Chinese herbal medicine in aquaculture has been reported in *Litopenaeus vannamei* [10], *Pelteobagrus fulvidraco* [11], *Barchydanio rerio var* [12] and *Oreochromis niloticus* [13]. Chinese herbal medicine can promote growth, enhance immunity and improve intestinal flora [14,15,16].

Chinese herbal medicine can be added to feed in two ways: The first is in the form of a single traditional Chinese medicine. The effect of this addition has been found to be biased and can only exert partial efficacy. The second way is in the form of a combination of multiple Chinese herbal medicines with different functions. This compound form of Chinese herbal medicine can simultaneously perform multiple functions and is more efficient and comprehensive than single Chinese herbs. At present, the application of Chinese herbal compound prescriptions in fisheries is increasing. However, so far, there are no reports on the application of *Lycium barbarum*, *Panax ginseng* and *Phragmitis rhizoma* as feed additives in the breeding of *M. amblycephala*. Therefore, in this study, four Chinese herbal medicines—*Astragalus membranaceus*, *Lycium barbarum*, *Panax ginseng* and *Rhizoma phragmitis*—were prepared in a compound Chinese herbal medicine, following the principle of “monarch, minister, assistant and envoy” in “*Huangdi Neijing*” [17]. This compound Chinese herbal medicine was first added to the basic feed of *M. amblycephala*, and breeding experiments were performed to explore its effects on the growth, immunity and digestive function of *M. amblycephala*, so as to provide a valuable basis for the green and healthy breeding of this species.

## 2. Materials and Methods

### 2.1. Experimental Materials

The experimental *M. amblycephala* (42 ± 2 g) were taken from the aquaculture breeding farm of Hunan University of Arts and Science. Before sampling, the experimental fish were temporarily cultured in the laboratory circulating culture system for two weeks. The radius and height of the round plastic culture tank were 1 m and 1.5 m, respectively. During the culture process, oxygen was aerated for 24 h to maintain an appropriate dissolved oxygen level of 6.0 ± 0.2 mg/L. The water temperature was 28 ± 2 °C. Carp adult fish extruded feed (Guangdong Tongwei Feed Co., Ltd., Guangzhou, China), which contained 31% protein, was fed to the experimental fish at 2% of their body weight twice a day.

### 2.2. Preparation and Application of Compound Chinese Herbal Medicine Feed

*Astragalus membranaceus* was purchased from Beijing Tongrentang Xing’an Health Care Technology Co., Ltd. (Beijing, China), *Lycium barbarum* was purchased from Ningxia Zhongning Goji Industry Development Co., Ltd. (Zhongning, China), *Panax ginseng* was purchased from Fusong County Changbai Mountain Tongda Ginseng Production and Sales Co., Ltd. (Jilin, China) and *Phragmitis rhizoma* was purchased from Baoji Liupan Yun Biological Technology Co., Ltd. (Baoji, China).

*Astragalus membranaceus*, *Lycium barbarum*, *Panax ginseng* and *Phragmitis rhizoma* were ground into powder and mixed according to a ratio of 4.5:3:1.5:1 to obtain the compound Chinese herbal medicine. The composition and nutritional levels of this compound medicine are shown in Table 1. It was added to the basic feed of *M. amblycephala* in different concentration gradients: 0% (control group, C), 1% (T1), 2% (T2) and 4% (T3). The composition and nutritional levels of the feed for the experimental groups are shown in Table 2. Three parallel culture tanks were used for each group, with thirty *M. amblycephala* placed in each tank. After two weeks of temporary rearing and domestication, the experimental fish were fed with feed containing different concentrations of the compound Chinese herbal medicine at 8:30 a.m. and 17:00 p.m. At each feed, the fish were given 2% of their weight in food; the body weight of the experimental fish was measured weekly to adjust rations. One third of the total volume of the aquaculture water was changed once a day, and the pollution in the culture tank was removed. The culture tank was aerated with oxygen for 24 h, and the culture was maintained for 90 days.

### 2.3. Determination of Growth Indicators

At the beginning of the experiment, the initial weight of each fish was measured and the average weight (W0) and total weight (WT0) were calculated. After 90 days of feeding, the total feed consumption (TFC) was calculated, and the final weight of each fish was measured. The average final weight (W1) and total weight (WT1) were then calculated.

Weight gain rate (WGR) = (W1 − W0)/W0 × 100%

Specific growth rates (SGR) = (lnW1 − lnW0)/T × 100%

Feed Conversion Ratio (FCR) = TFC/(WT1 − WT0)

Each treatment group was placed in three parallel repeated tanks, with 30 *M. amblycephala* in each tank. After 90 days of feeding, five *M. amblycephala* were taken from each tank. As there were three parallel tanks, 15 fish from each group were weighed (Wn). After dissection, the liver weight (Ln) and spleen weight (Sn) of the 15 fish were measured. Based on the corresponding body weights of these 15 fish, the liver and spleen body indices of each fish were calculated. Then, the average values were calculated.

Hepatosomatic index (HSI) = (Ln1/Wn1 + Ln2/Wn2 + Ln3/Wn3…… + Ln15/Wn15)/15 × 100%

Spleen index (SPI) = (Sn1/Wn1 + Sn2/Wn2 + Sn3/Wn3…… + Sn15/Wn15)/15 × 100%

### 2.4. Observation of Liver and Intestinal Tissue Sections

A compound Chinese herbal medicine was added to the basic feed of *M. amblycephala*. After 90 days of feeding, five *M. amblycephala* were taken from each tank. As there were three parallel tanks, 15 fish were sampled in each group. After inducing anesthesia with MS-222 (100 mg/L), blood was drawn from the tail vein via a syringe, and then the intestine (0.5 cm) and liver (~0.25 cm^2^) were removed and fixed in 4% neutral buffered paraformaldehyde for 48 h. The fixed samples were dehydrated with 50%, 70%, 80%, 90% and 95% ethanol and twice with anhydrous ethanol for 30 min, then cleaned in a xylene–ethanol solution for 2 h and pure xylene for 2 h twice. The tissue samples were soaked in three paraffin tanks in turn, each for 1 h, then embedded in paraffin. Pre-cooled wax blocks were fixed on a rotary microtome and cut into sections with a thickness of 5–7 µm. After cutting the first section, ten sections were cut continuously and discarded, and the eleventh section was selected. The two selected sections were separated by 10 sections. The sections were stained using the conventional hematoxylin–eosin (HE) staining method. Hepatic sinusoids in liver and structural changes of intestinal tissue were observed under an Olympus microscope (Olympus, Tokyo, Japan). Thickness of intestinal muscle layer and length and width of intestinal villi were detected by the CaseViewer 2.4 software. Three parallel repeated sections of the liver and middle intestine per individual were analyzed.

### 2.5. Determination of Serum Antioxidant and Immune-Related Enzyme Activities

Five *M. amblycephala* were taken from each parallel tank, resulting in 15 fish sampled from each group. A total of 1 mL of blood was taken from the tail vein of each *M. amblycephala* via sterile syringe and was centrifuged at 4000 r/min for 10 min at 4 °C to separate the serum. The serum of every fifth fish was mixed as a sample. The lysozyme (Lys) contents and the activities of total superoxide dismutase (T-SOD), catalase (CAT), acid phosphatase (ACP), alkaline phosphatase (AKP) and glutathione peroxidase (GSH-PX) were detected using Lys, T-SOD, CAT, ACP, AKP and GSH-PX detection kits, respectively, according to the manufacturer’s instructions. These kits were purchased from the Nanjing Jiancheng Institute of Bioengineering (Nanjing, China). The absorbance of Lys, T-SOD, CAT, ACP, AKP and GSH-PX was detected at 530 nm, 550 nm, 405 nm, 520 nm, 520 nm and 412 nm, respectively, using a multi-function microplate reader (BioTek, Winooski, VT, USA). The content of Lys is represented in μg/mL, and the activities of T-SOD, CAT, ACP, AKP and GSH-PX are represented in U/mL.

### 2.6. Determination of Enzyme Activity Related to Intestinal Digestion

Fifteen fish were sampled in each group. After anesthesia with MS-222 and following blood collection, 2 g of intestinal tissue was taken from each *M. amblycephala*, respectively, then precooled physiological saline (4 °C) was added according to the ratio of 1:9 (*w*/*v*). The tissue was then ground into homogenate in an ice water bath. The homogenate was centrifuged at 3000 r/min for 10 min at 4 °C to separate the supernatant. The supernatant of every fifth fish was mixed as a sample. The activities of amylase (AMS), lipase (LPS), and trypsin (TRS) in the intestines of *M. amblycephala* were detected using AMS, LPS and TRS detection kits, respectively, according to the manufacturer’s instructions using a multi-function microplate reader (BioTek, USA). The kits were purchased from Nanjing Jiancheng Institute of Bioengineering.

### 2.7. Mortality Statistics of M. amblycephala After Aeromonas hydrophila Infection

*Aeromonas hydrophila* was isolated from diseased *M. amblycephala*. The monoclonal strain was selected and it was inoculated in Luria broth (LB) liquid medium and shake-cultured overnight at 28 °C. In the pre-experiment, we diluted *A. hydrophila* to five different concentrations: 1.0 × 10^5^ CFU/mL, 1.0 × 10^6^ CFU/mL, 1.0 × 10^7^ CFU/mL, 1.0 × 10^8^ CFU/mL and 1.0 × 10^9^ CFU/mL. A total of 0.1 mL *A. hydrophila* at different concentrations was intraperitoneally injected into *M. amblycephala*. The control group was injected with 0.1 mL of sterilized PSB solution. Each group was placed in three parallel repeated tanks with 30 *M. amblycephala* in each tank. The cumulative mortality rates were determined for 96 h after infection. The mortality rates of the control group and the groups injected with different concentrations of *A. hydrophila* were 0, 13%, 35%, 51%, 65% and 85%, respectively. A curve was drawn, with the concentration of *A. hydrophila* as the abscissa and the cumulative mortality at 96 h as the ordinate. According to the linear interpolation method [18], the semi-lethal concentration of *A. hydrophila* against *M. amblycephala* at 96 h was 1.0 × 10^7^ CFU/mL. *M. amblycephala* were fed with either 0 (control group), 1%, 2% or 4% compound Chinese herbal medicine for 90 days, and then 0.1 mL (1 × 10^7^ CFU/mL) of *A. hydrophila* was injected intraperitoneally to infect *M. amblycephala*. The cumulative mortality rates of *M. amblycephala* in different experimental groups at 1d, 3d, 5d, 7d and 14d after infection were statistically analyzed.

### 2.8. Detection of the Expression of Immune-Related Genes in M. amblycephala by qRT-PCR

The basic feed of *M. amblycephala* was supplemented with 1%, 2% and 4% compound Chinese herbal medicine and cultured for 90 days. Five *M. amblycephala* were taken from each parallel tank, resulting in 15 fish sampled from each group. After being anesthetized by MS-222 and following the removal of blood from the tail vein, 100 mg of head kidney, liver, spleen, intestine and gill was taken from *M. amblycephala* and ground into a powder in a mortar using liquid nitrogen. Using an RNA extraction kit (Simgen, Hangzhou, China), according to the manufacturer’s instructions, RNA was extracted from the tissues of *M. amblycephala*. After agarose gel electrophoresis, the total RNA of each tissue exhibited three complete bands with no impurity bands: bands at 28S and 18S, which were clear with a brightness ratio of approximately 2:1, and a band at 5.8S, which was faintly visible. Moreover, the A260/280 ratio of the total RNA of each tissue was between 1.8 and 2.0, indicating that the quality of RNA met the requirements of the experiment. First-strand cDNA was synthesized using a PrimeScript^®^ RT reagent kit with gDNA Eraser (TaKaRa, Osaka, Japan) according to the manufacturer’s instructions.

Three reference genes—β-actin, 18S rRNA, and EF1a (elongation factor 1, alpha)—were selected based on expression stability; all primer sequences are detailed in Table 3. Products of the qRT-PCR primers were sequenced to confirm specificity. To select the reference genes with the most stable expression, their relative stability measure (M) was calculated using GeNorm (http://genorm.cmgg.be/, accessed on 12 March 2026), as described in previous studies [19]. M represents the average pairwise variation of a reference gene with all other reference genes; a lower M corresponds to higher expression stability [20]. The M values of reference genes decreased in the order EF1a > 18S rRNA > β-actin. According to this order, the most stable gene in the analyzed samples was β-actin, which is in line with our previous studies [21]. In addition, the PCR amplification efficiency of β-actin is much more similar to the immune-related genes of *M. amblycephala* (Table 3).

qRT-PCR was carried out in triplicate on a Rotor-Gene Q real-time PCR Detection System (QIAGEN, Dusseldorf, Germany) using the SYBR^®^ Premix Ex Taq™ II (TaKaRa, Japan) according to the manufacturer’s instructions. The total reaction volume of 20 μL contained 10 μL of SYBR qPCR Mix, 1 μL of each primer (10 mM), 2 μL of cDNA and 6 μL of ddH_2_O. The amplification conditions were pre-degeneration at 95 °C for 2 min; degeneration at 94 °C for 30 s; annealing at 56 °C for 30 s; extension at 72 °C for 30 s, 40 cycles; 72 °C for 8 min; and termination at 4 °C. The PCR reaction without a DNA sample was used as a control. The qRT-PCR specificity was verified via melting curve analysis. Gene expression values were calculated as fold-changes in the target gene relative to the reference gene (β-actin): fold change = 2^−ΔΔCt^, where ΔΔCt = (Ct target gene − Ct β-actin) [22].

### 2.9. Data Analysis

The experimental data are expressed as means ± standard error (M ± SE) and were subjected to a one-way analysis of variance (ANOVA) followed by Duncan’s test to determine differences among treatments. Differences were considered significant at *p* < 0.05. Statistical analysis was performed using SPSS15.0.

## 3. Results

### 3.1. Changes in Growth Indices of M. amblycephala

**Figure 1:**
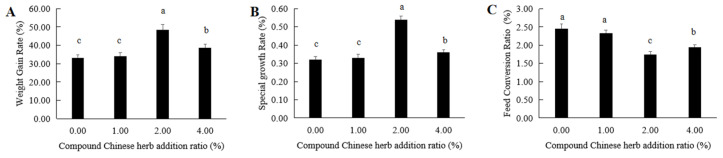
Effects of compound Chinese herbal medicine on the growth performance of *M. amblycephala*. (**A**), weight gain rate; (**B**), specific growth rate; (**C**), feed conversion ratio. The same lowercase letters indicate no significant difference (*p* > 0.05). Different lowercase letters indicate significant differences (*p* < 0.05).

The weight gain rates of *M. amblycephala* fed with basic feed supplemented with compound Chinese herbal medicine for 90 days were 33.15%, 34.18%, 48.40% and 38.78% in the control group, T1, T2 and T3 experimental groups, respectively (Figure 1A). In the control group and T1, T2 and T3 experimental groups, the specific growth rates of *M. amblycephala* were 0.32%, 0.33%, 0.54% and 0.36%, respectively (Figure 1B). Both the weight gain and specific growth rates in each experimental group were higher than those of the control group. These rates were highest in the T2 experimental group (Figure 1A,B). The feed conversion ratios in the control group and T1, T2 and T3 experimental groups were 2.45, 2.33, 1.75 and 1.95, respectively (Figure 1C). The feed coefficient was the lowest in the T2 experimental group (Figure 1C).

### 3.2. Changes in Intestinal Digestive Enzyme Activities

**Figure 2:**
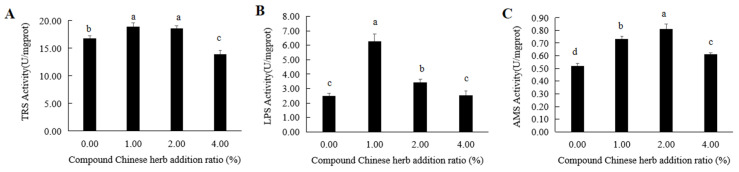
Effects of compound Chinese herbal medicine on the intestinal digestive enzyme activity of *M. amblycephala*. (**A**), intestinal trypsin activity; (**B**), intestinal lipase activity; (**C**), intestinal amylase activity. The same lowercase letters indicate no significant difference (*p* > 0.05). Different lowercase letters indicate significant differences (*p* < 0.05).

In the control group and T1, T2 and T3 experimental groups, the activities of trypsin (TRS) were 67.74 U/mgprot, 18.95 U/mgprot, 18.60 U/mgprot and 13.97 U/mgprot, respectively (Figure 2A), and the activities of lipase (LPS) were 2.56 U/mgprot, 6.26 U/mgprot, 3.41 U/mgprot and 2.53 U/mgprot, respectively (Figure 2B), in the intestine of *M. amblycephala*. In the T1 and T2 experimental groups, these activities were significantly higher than those in the control group (Supplementary Table S1), and they were highest in the T1 experimental group (Figure 2A,B). In the T3 experimental group, the intestinal TRS activity of *M. amblycephala* was significantly lower than that of the control group (Supplementary Table S1), but there was no significant difference in intestinal LPS activity (Supplementary Table S1, Figure 2A,B). The amylase (AMS) activities in the intestine of *M. amblycephala* in the control group and T1, T2 and T3 experimental groups were 0.52 U/mgprot, 0.73 U/mgprot, 0.81 U/mgprot and 0.61 U/mgprot, respectively (Figure 2C). These activity values were significantly higher in the T1, T2 and T3 experimental groups than in the control group (Supplementary Table S1, Figure 2C). The intestinal AMS activity was the highest in the T2 experimental group (Figure 2C).

### 3.3. The Effect of Compound Chinese Herbal Medicine on the Intestines of M. amblycephala

**Figure 3:**
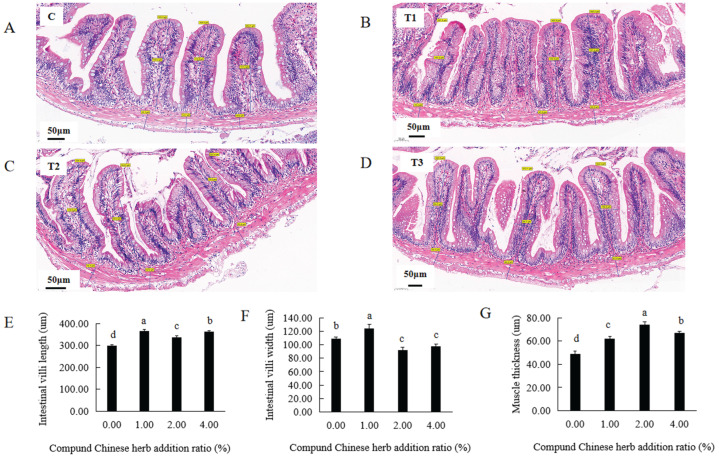
Effects of compound Chinese herbal medicine on the intestinal tissue of *M. amblycephala*. (**A**), control group intestine; (**B**), T1 experimental group intestine; (**C**), T2 experimental group intestine; (**D**), T3 experimental group intestine; (**E**), length of intestinal villi; (**F**), width of intestinal villi; (**G**), thickness of intestinal muscle layer. The blue lines indicate the measured thickness of intestinal wall, width and length of intestinal villi. The same lowercase letters indicate no significant difference (*p* > 0.05). Different lowercase letters indicate significant differences (*p* < 0.05).

In the T1, T2 and T3 experimental groups, the length of the intestinal villi in *M. amblycephala* was 364.37 μm, 336.80 μm and 363.20 μm, respectively, significantly higher than that in the control group (Supplementary Table S1, Figure 3A–E). In addition, the thickness of the intestinal muscle layer in these groups was 61.97 μm, 74.00 μm and 66.93 μm, respectively, significantly thicker than that of the control group (Supplementary Table S1, Figure 3A–D,G). The intestinal villi were longest in the T1 experimental group and the intestinal muscle layer was thickest in the T2 experimental group (Figure 3A–E,G). The width of intestinal villi in the T1 experimental group was 123.77 μm, significantly wider than that in the control group (Supplementary Table S1, Figure 3A–D,F), while in the T2 and T3 experimental groups, this width was 91.63 μm and 97.67 μm, respectively, significantly lower than that in the control group (Supplementary Table S1, Figure 3A–D,F).

### 3.4. The Effect of Compound Chinese Herbal Medicine on the Liver and Spleen of M. amblycephala

**Figure 4:**
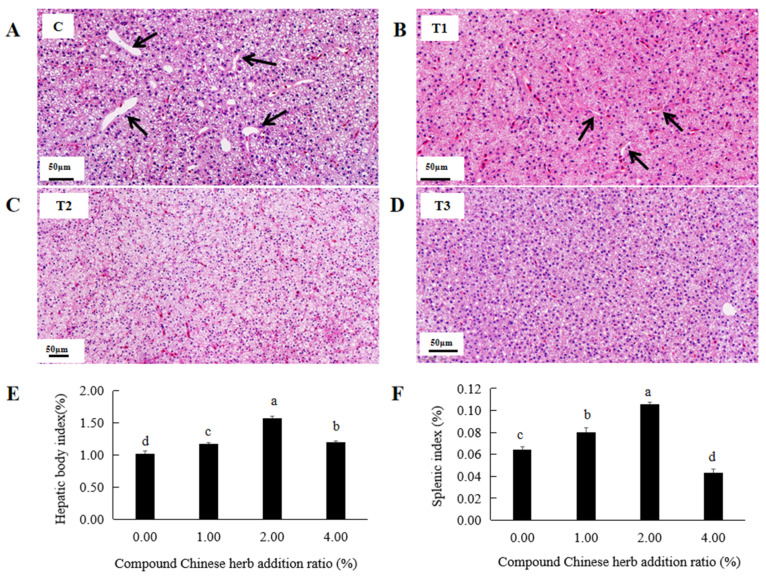
Effects of compound Chinese herbal medicine on the liver and spleen of *M. amblycephala*. (**A**), control liver; (**B**), T1 experimental group liver; (**C**), T2 experimental group liver; (**D**), T3 experimental group liver; (**E**), liver body index; (**F**), spleen body index. The black arrows indicate the hepatic sinus space. The same lowercase letters indicate no significant difference (*p* > 0.05). Different lowercase letters indicate significant differences (*p* < 0.05).

A large number of dilated hepatic sinusoids were detected in the control group, while this number significantly decreased in the T1 experimental group (Figure 4A,B). In the T2 and T3 experimental groups, dilated hepatic sinusoids were not detected (Figure 4C,D). The liver body indices of *M. amblycephala* in the T1, T2 and T3 experimental groups were 1.17%, 1.57% and 1.20%, respectively, significantly higher than that in the control group (Supplementary Table S1, Figure 4E). In the T1 and T2 experimental groups, the spleen body indices of *M. amblycephala* were 0.08% and 0.11%, respectively, significantly higher than that of the control group (Supplementary Table S1, Figure 4F). Moreover, the spleen and liver body indices were highest in the T2 experimental group (Figure 4E,F). However, in the T3 group, the spleen body index of *M. amblycephala* was 0.04%, significantly lower than that of the control group (Supplementary Table S1, Figure 4F).

### 3.5. The Effect of Compound Chinese Herbal Medicine on the Activity of Serum Immune-Related Enzymes

**Figure 5:**
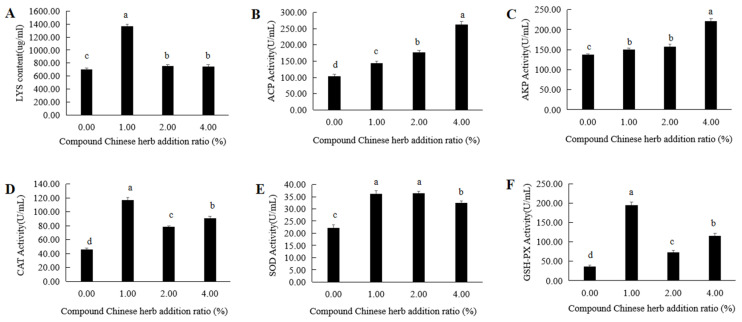
Effects of compound Chinese herbal medicine on the activity of serum immune-related enzymes of *M. amblycephala*. (**A**). Lysozyme content; (**B**), ACP activity; (**C**), AKP activity; (**D**), CAT activity; (**E**), SOD activity; (**F**), GSH-PX activity. The same lowercase letters indicate no significant difference (*p* > 0.05). Different lowercase letters indicate significant differences (*p* < 0.05).

In the T1, T2 and T3 experimental groups, the lysozyme (Lys) contents in the serum of *M. amblycephala* were 1365.03 µg/mL, 752.68 µg/mL and 745.36 µg/mL, respectively, significantly higher than that of the control group (Supplementary Table S1, Figure 5A). The activities of acid phosphatase (ACP) in the T1, T2 and T3 experimental groups were 143.79 U/mL, 177.51 U/mL and 263.31 U/mL, respectively, significantly higher than that of the control group (Supplementary Table S1, Figure 5B), and the activities of alkaline phosphatase (AKP) in these groups were 149.87 U/mL, 156.75 U/mL and 220.95 U/mL, respectively, again, significantly higher than that of the control group (Supplementary Table S1, Figure 5C). The activities of catalase (CAT) were 116.93 U/mL, 78.29 U/mL and 90.84 U/mL, respectively; those of total superoxide dismutase (T-SOD) were 36.27 U/mL, 36.42 U/mL and 32.38 U/mL, respectively; and those of glutathione peroxidase (GSH-PX) were 195.16 U/mL, 73.85 U/mL and 116.04 U/mL, respectively, in the T1, T2 and T3 experimental groups. All these values were significantly higher than that of the control group (Supplementary Table S1, Figure 5D–F). The Lys content and the activities of T-SOD, CAT and GSH-PX in the serum of *M. amblycephala* were the highest in the T1 experimental group (Figure 5A,D–F). The ACP and AKP activities in the serum of *M. amblycephala* gradually increased with the increase in amount of compound Chinese herbal medicine added to the feed, and thus, they were the highest in the T3 experimental group (Figure 5B,C).

### 3.6. The Effect of Compound Chinese Herbal Medicine on the Expression of Immune-Related Genes

**Figure 6:**
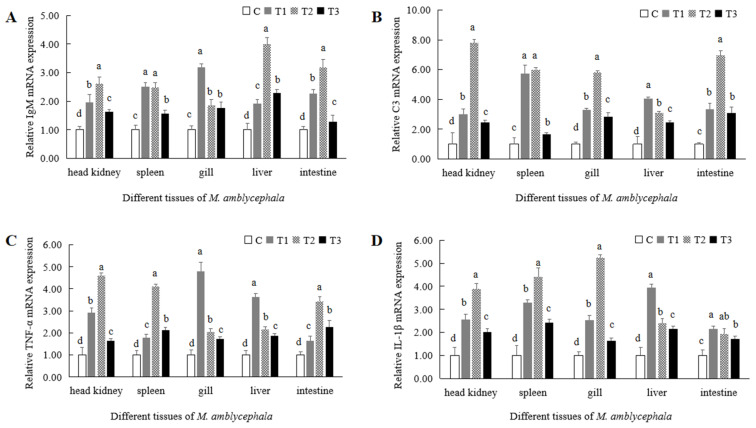
Effects of compound Chinese herbal medicine on the expression of immune-related genes of *M. amblycephala*. (**A**), relative expression levels of IgM; (**B**), relative expression levels of C3; (**C**), relative expression levels of TNF-α; (**D**), relative expression levels of IL-1β. The same lowercase letters indicate no significant difference (*p* > 0.05). Different lowercase letters indicate significant differences (*p* < 0.05).

In the experimental groups, the expression levels of IgM, C3, TNF- α and IL-1β in the head kidney, spleen, gills, liver and intestine of *M. amblycephala* were significantly higher than those in the control group (Supplementary Table S1, Figure 5). In the head kidney, liver and intestine, the IgM expression levels were the highest in the T2 experimental group (Figure 6A), while in the spleen and gills, they were the highest in the T1 experimental group (Figure 6A). The C3 expression levels in the head kidney, spleen, gills and intestine were the highest in the T2 experimental group (Figure 6B), while it was the highest in the liver in the T1 experimental group (Figure 6B). In the head kidney, spleen and intestine, the TNF-α expression levels were the highest in the T2 experimental group, while in the gills and liver, they were the highest in the T1 experimental group (Figure 6C). The IL-1β expression levels in the head kidney, spleen and gills were the highest in the T2 experimental group, while they were highest in the liver and intestine in the T1 experimental group (Figure 6D).

### 3.7. The Effect of Compound Chinese Herbal Medicine on Disease Resistance of M. amblycephala

**Figure 7:**
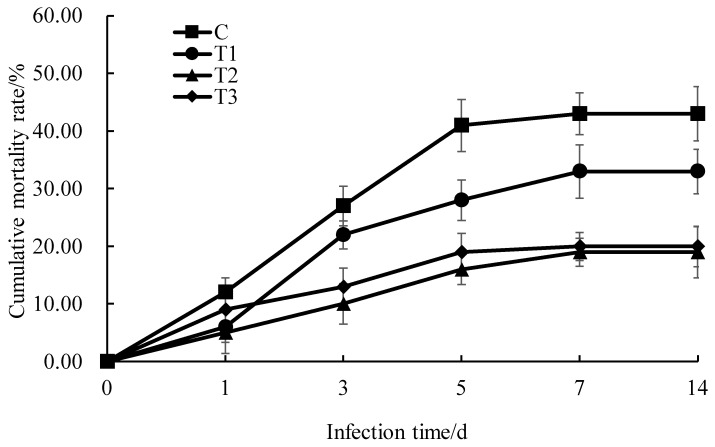
Effect of compound Chinese herbal medicine on the cumulative mortality rate of *M. amblycephala* after infection with *A. hydrophila*.

After infection with *A. hydrophila*, the cumulative mortality of *M. amblycephala* in the control group and T1, T2 and T3 experimental groups gradually increased, reaching a peak of 53%, 33%, 19% and 20%, respectively, on the 7th day after infection (Figure 7). In the experimental groups, the mortality of *M. amblycephala* infected with *A. hydrophila* was significantly lower than that of the control group (Supplementary Table S1). On the 7th day of infection with *A. hydrophila* in *M. amblycephala*, there was no significant difference in cumulative mortality between the T2 and T3 experimental groups (Supplementary Table S1). The cumulative mortality rate was lowest in the T2 experimental group, which was fed with 2% compound Chinese herbal medicine feed (Figure 7).

## 4. Discussion

Adding a compound Chinese herbal medicine composed of *Astragalus memeranaceus*, *Lycium barbarum*, *Panax ginseng* and *Phragmitis rhizoma* to the basic feed of *M. amblycephala* significantly increased the weight gain rate and specific growth rate and reduced the feed conversion ratio. Chinese herbal medicine is rich in active ingredients and nutrients, which could help promote growth and improve muscle quality. After the addition of 0.4% of a compound preparation consisting of 12 Chinese herbal medicines, including *Codonopsis pilosula*, *Astragalus memeranaceus* and *Isatis tinctoria*, into the feed of *Acanthopagrus schlegelii*, the weight gain rate and specific growth rate significantly improved, the feed coefficient reduced and the growth performance improved in the experimental group [23]. The compound Chinese herbal medicine composed of *Astragalus memeranaceus*, *Isatis tinctoria*, *Crataegus pinnatifida* and *Angelica sinensis* could significantly improve the growth performance of pearl gentian grouper (*Epinephelus lanceolatus* ♂ × *Epinephelus fuscoguttatus* ♀), reporting that the optimal addition amount was 1.5% [24]. However, in the present study, the optimal addition amount was 2%, which indicates that different Chinese herbal medicines have different synergistic effects. These differences in Chinese herbal medicine types, or differences in fish species, may cause the variation in optimal addition amounts.

After adding a compound Chinese herbal medicine preparation composed of *Astragalus memeranaceus*, *Lycium barbarum*, *Panax ginseng* and *Phragmitis rhizoma* in amounts of 1% and 2% to the basic feed of *M. amblycephala*, the activities of trypsin, amylase and lipase in the intestine of *M. amblycephala* were significantly higher than those in the control group. Moreover, in this species, the weight gain and specific growth rates were the highest and the feed conversion ratio was the lowest in the experimental group supplemented with 2% compound Chinese herbal medicine. Adding this compound Chinese herbal medicine to the basic feed of *M. amblycephala* could significantly improve the growth performance, which was in line with previous studies [25,26,27]. These results indicate that some active ingredients in the compound Chinese herbal medicine might enhance enzyme activity in the intestine of *M. amblycephala*, as well as improve its digestion and feed absorption rate, thus promoting growth. However, the mechanism of this action and the compatibility principle still require further research.

In fish, the liver provides a variety of functions such as metabolism, digestion, detoxification and immunity [28]. Chinese herbal medicines are rich in polysaccharides, amino acids and trace elements, which can improve the palatability of feed, increase feed intake and nutrient digestibility [14]. In this study, in experimental groups supplemented with compound Chinese herbal medicine, the liver body indices were significantly higher than that in the control group, which was regarded as a sign of “improved nutritional status and accelerated growth”.

*Codonopsis pilosula* had a protective effect on the liver of *Acipenser schrenckii* [29]. Extracts of *Angelica sinensis*, *Ginkgo biloba* and *Scutellaria baicalensis* also have significant positive effects on liver tissue injury and integrity, which could lead to restoration of oxidase activity and down-regulation in the expression of inflammatory factors, protecting the liver [30]. In this study, a large number of dilated hepatic sinusoids were detected in the control group, while this number was significantly decreased in the T1 experimental group and not detected in the T2 and T3 experimental groups, which indicates that adding compound Chinese herbal medicine to the basic feed of *M. amblycephala* could have protective effects for the liver.

The intestine is an important mucosal-associated lymphoid tissue (MALT) in teleost fish, and the intestinal mucosal layer plays an important role in the mucosal immune function of this organ [31]. After feeding *Litopenaeus vannamei* with a compound preparation of Chinese herbal medicine composed of *Nandina domestica* and *Prunus mume* for 30 days, there was no obvious damage to their intestinal tissue [32]. In this study, in experimental groups of *M. amblycephala* supplemented with a compound Chinese herbal medicine composed of *Astragalus memeranaceus*, *Lycium barbarum*, *Panax ginseng* and *Phragmitis rhizoma*, the length of the intestinal villi and the thickness of the intestinal muscle layer were significantly higher than those of the control group, which was similar to the results of a previous study [33]. Moreover, in the experimental group supplemented with 1% of this compound, the length and width of intestinal villi were the highest, while the thickness of the intestinal muscle layer was the highest in the experimental group supplemented with 2% of this compound. This indicates that addition of this compound to the basic feed of *M. amblycephala* could improve its intestinal tissue structure.

The addition of a compound Chinese herbal medicine preparation consisting of *Astragalus memeranaceus*, *Lycium barbarum*, *Panax ginseng* and *Phragmitis rhizoma* to the basic feed of *M. amblycephala* significantly increased the serum Lys content and the activities of T-SOD, CAT, ACP, AKP and GSH-PX compared to the control group. Chinese herbal medicines contain various immune-active substances such as alkaloids, organic acids and polysaccharides, which can enhance the phagocytic ability of phagocytes, promote metabolism and improve antioxidant capacity, thus improving immunity [34]. After adding a compound Chinese herbal medicine preparation to the basal feed of grouper, the total protein (TP) content and the activities of AKP, ACP and T-SOD in serum all significantly increased [35]. Furthermore, at addition ratios of 0.8% and 1.2%, the immune ability of the groupers was significantly improved [35]. The activities of LZM, AKP, SOD and ACP in *Amphiprion frenatus* serum significantly increased after adding different doses of compound Chinese herbal medicine [36]. The best effect was achieved when the dosage was 20 g/kg, with significant improvements in the immune ability of *Amphiprion frenatus* observed [36]. In the serum of Japanese eels, the activities of non-specific immune enzymes such as SOD, AKP and Lys significantly increased following the addition of a compound Chinese herbal medicine containing *Rheum palmatum*, *Glycyrrhiza uralensis* and *Astragalus membranaceus* to their basic feed [37]. In this study, the activities of ACP and AKP gradually increased with the increase in the amount of the compound Chinese herbal medicine, peaking at an addition amount of 4%. Lys contents and T-SOD, CAT and GSH-PX activities were highest in the experimental group supplemented with 1% of this compound, which indicates that this compound Chinese herbal medicine could significantly increase the activities of non-specific immune-related enzymes in the serum of *M. amblycephala*.

Adding a compound Chinese herbal medicine composed of *Astragalus memeranaceus*, *Lycium barbarum*, *Panax ginseng* and *Phragmitis rhizoma* to the basic feed of *M. amblycephala* significantly increased the IgM and C3 expression. IgM was the first immunoglobulin discovered in bony fish and plays an important role in resisting pathogen invasion and infection. The content of IgM in the serum of hybrid snake head fish (*Channa maculate* × *Channa argus*) significantly increased following supplementation of its feed with *Astragalus memeranaceus* polysaccharide [38]. Complement C3 is an important component of the complement system that plays a significant role in the immune system of fish. The feed of *Pelteobagrus fulvidraco* was supplemented with Chinese herbal medicines such as *Codonopsis pilosula*, *Astragalus membranaceus* and *Chrysanthemi indici*, which was shown to significantly increase the content of serum complement C3 [39]. In rainbow trout, compound Chinese herbal medicine significantly increased the serum C3 content [40]. Adding 1.0% and 1.5% compound Chinese herbal medicine to feed also increased the contents of complement C3 and IgM in the serum of *Acipenser dabryanus* [41]. In this study, the expression levels of IgM and C3 in the head kidney, spleen, gills, liver and intestines of *M. amblycephala* were significantly higher in the experimental groups supplemented with the compound Chinese herbal medicine than those in the control group. This indicates that this compound Chinese herbal medicine could induce IgM and C3 expression, which might have a promoting effect on the immune function of *M. amblycephala*.

The expression levels of interleukin-1β (IL-1β) and tumor necrosis factor α (TNF-α) significantly increased following the addition of a compound Chinese herbal medicine containing *Astragalus memeranaceus*, *Lycium barbarum*, *Panax ginseng* and *Phragmitis rhizoma* to their basic feed of *M. amblycephala.* IL-1β and TNF-α are pro-inflammatory cytokines, and their expression levels are important indicators of inflammatory response. After administration of feed containing 20 g/kg of compound Chinese herbal medicine, IL-1β, IL-8 and IFN-β were significantly up-regulated in rainbow trout [42]. Injection of *Astragalus memeranaceus* polysaccharide induced the expression of IL-1β in the head kidney of common carp, but it had no significant effect on the expression of IL-1β in the spleen or gills [43]. *Lentinus edodes* and *Astragalus memeranaceus* polysaccharides also significantly promoted the expression of IL-1β in the peripheral leukocytes of common carp [44]. Following feeding with dietary silymarin, IL-1β and TNF-α were up-regulated in hepatic tissues of *Oreochromis niloticus* [45]. Following the addition of 1.5% and 2.0% compound Chinese herbal medicine to the basal feed of *Oreochromis niloticus*, the expression of TNF-α and IL-1β significantly increased in the liver, gills, thymus, head kidney and spleen [46]. In this study, the expression of IL-1β and TNF-α in the head kidney, spleen, gills, liver and intestines of *M. amblycephala* was significantly higher in the experimental groups supplemented with compound Chinese herbal medicine compared to the control group. This indicates that this medicine could induce a pro-inflammatory reaction, which helped to improve the immunity and anti-stress ability of *M. amblycephala*.

After challenge by *A. hydrophila*, the mortality rate of *M. amblycephala* was significantly lower in the experimental groups supplemented with compound Chinese herbal medicine compared to the control group. Compound Chinese herbal medicines can enhance the immune function and reduce susceptibility to pathogenic microorganisms, which could have an important role in preventing fish diseases. Supplementing feed with compound Chinese herbal medicine enhanced the resistance of *Pelteobagrus fulvidraco* to artificial infection with *A. hydrophila* [47]. The survival rate of *Acipenser schrenckii* after being challenged by *A. hydrophila* was effectively improved by three kinds of compound Chinese herbal medicines prepared from *Artemisia scoparza*, *Radix isatidis*, *Rhei radix*, *Notoginseng radix*, *Radix arnebiae* and *Lonicera japonica* [48]. Following the addition of 1.0% and 1.5% compound Chinese herbal medicine to the basal feed of juvenile *Acipenser dabryanus*, their mortality rate after infection with *A. hydrophila* significantly decreased [41]. In this study, the cumulative mortality rate of *M. amblycephala* was lowest in the T2 experimental group, which was fed with 2% compound Chinese herbal medicine feed. This indicates that feeding this compound Chinese herbal medicine to fish could effectively enhance the immune function of *M. amblycephala* and increase resistance to *A. hydrophila*. The optimal addition amount for this compound Chinese herbal medicine in the basic feed for *M. amblycephala* was 2%.

## 5. Conclusions

The results of this study indicate that addition of the investigated compound Chinese herbal medicine to feed could significantly increase the expression levels of immune-related genes and the activity of immune-related enzymes in serum. It could also enhance disease resistance, improve intestinal and liver tissues, increase the activity of intestinal digestion-related enzymes and promote the growth of *M. amblycephala*. The appropriate addition amount of this compound Chinese herbal medicine in the basic feed of *M. amblycephala* was 2%. This compound Chinese herbal medicine preparation, as a natural and environmentally friendly feed additive, had a significant promoting effect on the growth of *M. amblycephala* and enhanced its immunity. However, the specific mechanism of action of this compound preparation requires further study in order to provide scientific basis for its wide application in aquaculture.

## Figures and Tables

**Table 1:** Composition and nutritional levels of the compound Chinese herbal medicine.

	*Astragalus membranaceus*	*Panax ginseng*	*Lycium barbarum*	*Phragmitis rhizoma*	Compound Chinese Herbal Medicine
Additive amount (%)	45	15	30	10	100
Crude Protein (%)	14.9	12.3	13.9	13	14.02
Crude fat (%)	1.1	0.1	5	2.3	2.24
Crude Ash (%)	5	5	6	3	5.1
Carbohydrate (%)	33.4	58.6	45	78	45.12
Cellulose (%)	42.1	17.1	16.9	1.4	26.72
Total energy (KJ/g)	9.8	13.20	13.14	17.6	12.1

**Table 2:** Composition and nutritional levels of the diets for the different experimental groups.

Ingredient	Experimental Groups			
	Control Group	T1	T2	T3
Soybean meal (g)	200	200	200	200
Rapeseed meal (g)	400	400	400	400
Fish meal (g)	110	110	110	110
Wheat flour (g)	175	165	155	135
Compound Chinese herbal medicine (g)	0	10	20	40
Soybean oil (g)	60	60	60	60
Compound feed premixes (g)	30	30	30	30
Bentonite (g)	23	23	23	23
Choline chloride (g)	1.7	1.7	1.7	1.7
Preservative (g)	0.1	0.1	0.1	0.1
Antioxidant (g)	0.2	0.2	0.2	0.2
Total (g)	1000	1000	1000	1000
Nutrient level				
Crude protein (%)	33.97	33.98	33.99	34.01
Crude fat (%)	8.85	8.86	8.86	8.88
Crude ash (%)	5.56	5.58	5.59	5.68
Calcium (%)	1.2	1.2	1.2	1.2
Carbohydrate (%)	29.83	29.59	29.35	28.88
Total phosphorus (%)	0.5	0.5	0.5	0.5
Crude fiber (%)	6.24	6.48	6.73	7.22
Total energy (MJ/kg)	16.75	16.71	16.68	16.60

**Table 3:** qRT-PCR primers for immune-related genes of *M. amblycephala.*

Primer Name	Sequence (5′–3′)	Accession Number	PCR Amplification Efficiency (%)	Slope	R^2^	Amplicon Size (bp)
il-1βF	ACCCACATGAAAACCTCCTGTT	MN294974.1	96	−3.483	0.996	215
il-1βR	GATCTGTTGTTTGTCCTCCAGC					
tnf-αF	CCGCTGCTGTCTGCTTCA	KU976426.1	95	−3.453	0.997	223
tnf-αR	GCCTGGTCCTGGTTCACTCT					
c3-F	ATGGACTTTCACTCGATCCAACA	MK165094.1	95	−3.476	0.994	258
c3-R	AACTGCTTCTCCATCTTCACACT					
igm-F	GGAGCAACGGCACAGTAT	KC894945	96	−3.481	0.995	221
igm-R	ATCAGCAAGCCAAGACAC					
β-actin-F	ACCCACACCGTGCCCATCTA	AY170122.2	95	−3.488	0.997	236
β-actin-R	CGGACAATTTCTCTTTCGGCTG					
18S rRNA-F	CGGAGGTTCGAAGACGATCA	AB860215.1	92	−3.450	0.991	247
18S rRNA-R	GGGTCGGCATCGTTTACG					
EF1a-F	CTTCTCAGGCTGACTGTGC	XM_048201754.1	90	−3.580	0.993	218
EF1a-R	CCGCTAGCATTACCCTCC					

## Data Availability

All data, models and code generated or used during the study are presented in the submitted article.

## Supplementary Materials

The following supporting information can be downloaded at: https://www.mdpi.com/article/10.3390/ani16060925/s1. Table S1. Results of one-way analysis of variance (ANOVA) among different experimental groups.
