# Supplementary material for: The Effects of Compound Chinese Herbal Medicine on the Growth and Digestive and Immune Systems of Megalobrama amblycephala
Source: Animals (Basel). 2026 Mar 15;16(6):925. doi: 10.3390/ani16060925 (PMC13023318; doi:10.3390/ani16060925)
Supplement: Supplementary file 1 [file animals-16-00925-s001.zip › Supplementary Table S1.pdf]

Supplementary Table S1. Results of one-way analysis of variance (ANOVA) among different experimental groups.

Parameters	Experimental groups	df	F	P
Weight gain rates	T1 vs C	1	2.253	0.152
	T2 vs C	1	16.542	8.408×10^{-4}
	T3 vs C	1	5.493	0.021
	T2 vs T1	1	20.385	5.408×10^{-4}
	T3 vs T1	1	7.412	0.016
	T3 vs T2	1	12.389	5.347×10^{-4}
Specific growth rates	T1 vs C	1	2.173	0.262
	T2 vs C	1	28.985	2.653×10^{-4}
	T3 vs C	1	8.549	0.032
	T2 vs T1	1	39.487	2.487×10^{-4}
	T3 vs T1	1	8.536	0.013
	T3 vs T2	1	35.246	2.356×10^{-4}
Feed Conversion Ratio	T1 vs C	1	2.328	0.165
	T2 vs C	1	26.658	1.324×10^{-4}
	T3 vs C	1	25.481	1.525×10^{-4}
	T2 vs T1	1	19..267	3.426×10^{-4}
	T3 vs T1	1	8.659	0.012
	T3 vs T2	1	7.862	0.022
Hepatosomatic index	T1 vs C	1	6.859	0.015
	T2 vs C	1	29.325	3.411×10^{-4}
	T3 vs C	1	7.736	0.025
	T2 vs T1	1	25.342	1.212×10^{-4}
	T3 vs T1	1	6.235	0.035
	T3 vs T2	1	28.563	1.423×10^{-4}
	T1 vs C	1	8.365	0.023
	T2 vs C	1	38.657	1.432×10^{-4}

Spleen index	T3 vs C	1	26.874	1.343×10^{-4}
	T2 vs T1	1	36.589	1.224×10^{-5}
	T3 vs T1	1	38.552	1.422×10^{-5}
	T3 vs T2	1	47.946	2.342×10^{-5}
Intestinal trypsin activity	T1 vs C	1	6.681	0.041
	T2 vs C	1	7.035	0.039
	T3 vs C	1	5.362	0.016
	T2 vs T1	1	1.863	0.334
	T3 vs T1	1	9.567	0.026
	T3 vs T2	1	9.551	0.023
Intestinal lipase activity	T1 vs C	1	67.285	3.314×10^{-4}
	T2 vs C	1	8.385	0.023
	T3 vs C	1	2.236	0.331
	T2 vs T1	1	53.412	2.121×10^{-5}
	T3 vs T1	1	63.582	3.124×10^{-5}
	T3 vs T2	1	7.822	0.021
Intestinal amylase activity	T1 vs C	1	39.456	1.422×10^{-5}
	T2 vs C	1	43.522	2.121×10^{-5}
	T3 vs C	1	6.533	0.031
	T2 vs T1	1	6.221	0.029
	T3 vs T1	1	33.421	1.121×10^{-4}
	T3 vs T2	1	56.341	2.312×10^{-5}
Length of intestinal villi	T1 vs C	1	59.736	2.341×10^{-5}
	T2 vs C	1	8.462	0.023
	T3 vs C	1	54.668	2.233×10^{-5}
	T2 vs T1	1	7.866	0.024
	T3 vs T1	1	7.224	0.032
	T3 vs T2	1	8.221	0.026
	T1 vs C	1	9.228	0.021

Width of intestinal villi	T2 vs C	1	8.228	0.019
	T3 vs C	1	8.771	0.017
	T2 vs T1	1	36.442	1.322×10^{-4}
	T3 vs T1	1	35.685	1.234×10^{-4}
	T3 vs T2	1	2.158	0.422
Thickness of intestinal muscle layer	T1 vs C	1	6.334	0.024
	T2 vs C	1	75.422	3.221×10^{-5}
	T3 vs C	1	8.655	0.035
	T2 vs T1	1	7.661	0.031
	T3 vs T1	1	6.658	0.025
	T3 vs T2	1	7.344	0.022
Lysozyme content	T1 vs C	1	121.825	1.109×10^{-6}
	T2 vs C	1	8.554	0.024
	T3 vs C	1	8.753	0.021
	T2 vs T1	1	110.287	1.056×10^{-6}
	T3 vs T1	1	108.766	1.043×10^{-6}
	T3 vs T2	1	1.711	0.447
ACP activity	T1 vs C	1	7.785	0.016
	T2 vs C	1	60.625	3.032×10^{-5}
	T3 vs C	1	145.663	1.223×10^{-6}
	T2 vs T1	1	6.558	0.023
	T3 vs T1	1	78.882	3.441×10^{-5}
	T3 vs T2	1	39.254	1.412×10^{-4}
AKP activity	T1 vs C	1	5.534	0.041
	T2 vs C	1	6.611	0.039
	T3 vs C	1	75.155	3.217×10^{-5}
	T2 vs T1	1	2.335	0.383
	T3 vs T1	1	58.336	2.411×10^{-5}
	T3 vs T2	1	47.857	2.342×10^{-4}

CAT activity	T1 vs C	1	243.521	1.217×10^{-6}
	T2 vs C	1	89.665	4.433×10^{-5}
	T3 vs C	1	97.842	4.342×10^{-6}
	T2 vs T1	1	58.441	2.421×10^{-5}
	T3 vs T1	1	33.224	1.156×10^{-4}
	T3 vs T2	1	8.776	0.013
SOD activity	T1 vs C	1	45.332	2.216×10^{-5}
	T2 vs C	1	44.581	2.229×10^{-5}
	T3 vs C	1	34.552	1.228×10^{-4}
	T2 vs T1	1	2.281	0.413
	T3 vs T1	1	8.447	0.017
	T3 vs T2	1	8.115	0.015
GSH-PX activity	T1 vs C	1	225.527	1.122×10^{-7}
	T2 vs C	1	7.884	0.021
	T3 vs C	1	8.225	0.016
	T2 vs T1	1	173.556	1.316×10^{-6}
	T3 vs T1	1	102.635	1.013×10^{-6}
	T3 vs T2	1	8.954	0.011
IgM expression in head kidney	T1 vs C	1	15.332	1.127×10^{-4}
	T2 vs C	1	21.653	2.038×10^{-4}
	T3 vs C	1	14.547	1.117×10^{-4}
	T2 vs T1	1	6.322	0.026
	T3 vs T1	1	5.134	0.047
	T3 vs T2	1	14.583	1.229×10^{-4}
IgM expression in spleen	T1 vs C	1	34.998	1.151×10^{-4}
	T2 vs C	1	35.257	1.213×10^{-4}
	T3 vs C	1	8.746	0.023
	T2 vs T1	1	2.144	0.465
	T3 vs T1	1	22.476	1.123×10^{-4}

	T3 vs T2	1	23.514	1.127×10^{-4}
IgM expression in gill	T1 vs C	1	43.582	2.129×10^{-5}
	T2 vs C	1	12.571	1.128×10^{-4}
	T3 vs C	1	11.226	1.016×10^{-4}
	T2 vs T1	1	15.247	1.112×10^{-4}
	T3 vs T1	1	15.884	1.144×10^{-4}
	T3 vs T2	1	2.113	0.466
IgM expression in liver	T1 vs C	1	8.774	0.022
	T2 vs C	1	125.437	1.127×10^{-6}
	T3 vs C	1	52.328	2.116×10^{-5}
	T2 vs T1	1	73.657	1.328×10^{-5}
	T3 vs T1	1	8.374	0.023
	T3 vs T2	1	45.657	2.327×10^{-4}
IgM expression in intestine	T1 vs C	1	34.258	1.212×10^{-4}
	T2 vs C	1	68.587	3.429×10^{-5}
	T3 vs C	1	8.654	0.021
	T2 vs T1	1	9.257	0.022
	T3 vs T1	1	12.542	1.127×10^{-4}
	T3 vs T2	1	28.557	1.422×10^{-4}
C3 expression in head kidney	T1 vs C	1	5.124	0.048
	T2 vs C	1	23.564	1.314×10^{-4}
	T3 vs C	1	4.986	0.049
	T2 vs T1	1	22.357	1.117×10^{-4}
	T3 vs T1	1	4.752	0.049
	T3 vs T2	1	21.546	1.027×10^{-4}
	T1 vs C	1	35.653	1.327×10^{-4}
	T2 vs C	1	37.845	1.342×10^{-4}
	T3 vs C	1	5.237	0.047

C3 expression in spleen	T2 vs T1	1	2.248	0.475
	T3 vs T1	1	25.647	1.232×10^{-4}
	T3 vs T2	1	28.541	1.427×10^{-4}
C3 expression in gill	T1 vs C	1	17.547	1.327×10^{-4}
	T2 vs C	1	29.364	1.418×10^{-4}
	T3 vs C	1	15.847	1.242×10^{-4}
	T2 vs T1	1	18.578	1.429×10^{-4}
	T3 vs T1	1	5.235	0.048
	T3 vs T2	1	15.286	1.214×10^{-4}
C3 expression in liver	T1 vs C	1	16.478	1.323×10^{-4}
	T2 vs C	1	8.792	0.024
	T3 vs C	1	6.457	0.045
	T2 vs T1	1	7.428	0.021
	T3 vs T1	1	14.581	1.229×10^{-4}
	T3 vs T2	1	7.847	0.027
C3 expression in intestine	T1 vs C	1	28.145	2.407×10^{-4}
	T2 vs C	1	148.543	1.242×10^{-6}
	T3 vs C	1	21.457	1.047×10^{-4}
	T2 vs T1	1	34.781	1.234×10^{-4}
	T3 vs T1	1	2.512	0.413
	T3 vs T2	1	33.285	1.114×10^{-4}
TNF-α expression in head kidney	T1 vs C	1	43.528	2.126×10^{-4}
	T2 vs C	1	112.537	1.012×10^{-6}
	T3 vs C	1	4.652	0.047
	T2 vs T1	1	17.854	1.019×10^{-3}
	T3 vs T1	1	11.247	1.023×10^{-4}
	T3 vs T2	1	89.263	4.413×10^{-5}
	T1 vs C	1	5.641	0.042

TNF-α expression in spleen	T2 vs C	1	63.571	3.128×10^{-5}
	T3 vs C	1	12.475	1.126×10^{-3}
	T2 vs T1	1	57.364	2.318×10^{-5}
	T3 vs T1	1	5.246	0.047
	T3 vs T2	1	34.358	1.217×10^{-4}
TNF-α expression in gill	T1 vs C	1	158.674	1.243×10^{-6}
	T2 vs C	1	14.325	1.216×10^{-3}
	T3 vs C	1	9.547	0.011
	T2 vs T1	1	89.546	4.427×10^{-5}
	T3 vs T1	1	96.248	4.312×10^{-5}
	T3 vs T2	1	6.141	0.048
TNF-α expression in liver	T1 vs C	1	78.647	3.432×10^{-5}
	T2 vs C	1	21.472	1.023×10^{-4}
	T3 vs C	1	11.486	1.025×10^{-3}
	T2 vs T1	1	23.571	1.126×10^{-4}
	T3 vs T1	1	26.843	1.324×10^{-4}
	T3 vs T2	1	5.281	0.045
TNF-α expression in intestine	T1 vs C	1	6.548	0.041
	T2 vs C	1	45.361	2.232×10^{-5}
	T3 vs C	1	18.713	1.435×10^{-4}
	T2 vs T1	1	28.547	1.427×10^{-4}
	T3 vs T1	1	6.234	0.046
	T3 vs T2	1	21.193	1.058×10^{-4}
IL-1β expression in head kidney	T1 vs C	1	11.342	1.017×10^{-3}
	T2 vs C	1	24.521	2.226×10^{-4}
	T3 vs C	1	9.526	0.011
	T2 vs T1	1	12.134	1.107×10^{-3}
	T3 vs T1	1	7.145	0.028
	T3 vs T2	1	20.361	1.019×10^{-4}

IL-1β expression in spleen	T1 vs C	1	31.278	1.063×10^{-4}
	T2 vs C	1	44.256	2.212×10^{-5}
	T3 vs C	1	11.352	1.117×10^{-3}
	T2 vs T1	1	9.472	0.012
	T3 vs T1	1	11.241	1.012×10^{-3}
	T3 vs T2	1	17.428	1.038×10^{-4}
IL-1β expression in gill	T1 vs C	1	14.781	1.239×10^{-3}
	T2 vs C	1	165.846	1.329×10^{-6}
	T3 vs C	1	9.472	0.011
	T2 vs T1	1	52.514	2.126×10^{-5}
	T3 vs T1	1	11.251	1.012×10^{-3}
	T3 vs T2	1	120.576	1.102×10^{-6}
IL-1β expression in liver	T1 vs C	1	47.814	2.239×10^{-5}
	T2 vs C	1	19.187	1.409×10^{-4}
	T3 vs C	1	13.256	1.123×10^{-3}
	T2 vs T1	1	18.254	1.412×10^{-4}
	T3 vs T1	1	21.146	2.007×10^{-4}
	T3 vs T2	1	5.141	0.049
IL-1β expression in intestine	T1 vs C	1	13.251	1.112×10^{-3}
	T2 vs C	1	9.471	0.012
	T3 vs C	1	8.132	0.017
	T2 vs T1	1	2.312	0.398
	T3 vs T1	1	6.851	0.042
	T3 vs T2	1	2.114	0.403
Cumulative mortality	T1 vs C	1	9.573	0.017
	T2 vs C	1	16.348	1.317×10^{-4}
	T3 vs C	1	14.581	1.229×10^{-3}
	T2 vs T1	1	8.864	0.026

	T3 vs T1	1	8.113	0.032
	T3 vs T2	1	2.581	0.352
